# Association between exposure to a PERMA model-based positive psychology intervention and levels of psychological distress and compliance behaviors in patients with type 2 diabetes mellitus: a quasi-experimental study

**DOI:** 10.3389/fpsyg.2026.1808234

**Published:** 2026-05-05

**Authors:** Tao Zeng, Kan Zhang, Wanjun Ding, Li Wen

**Affiliations:** 1 Endocrinology Department, Renmin Hospital of Wuhan University, Wuhan, Hubei, China; 2 Oncology Department, Renmin Hospital of Wuhan University, Wuhan, Hubei, China; 3 Nursing Department, Renmin Hospital of Wuhan University, Wuhan, Hubei, China

**Keywords:** PERMA mode, Type 2 diabetes mellitus, psychological distress, self-management, quasi-experimental study

## Abstract

**Objective:**

To explore the effect of positive psychological intervention of the PERMA model on psychological distress and compliance behavior in patients with type 2 diabetes mellitus (T2DM).

**Methods:**

This study used convenience sampling to enroll 120 patients with type 2 diabetes (T2DM) who were admitted between January and December 2023. Participants were assigned to one of two non-equivalent groups based on their treatment pathways in clinical practice: the intervention group (*n* = 60) that received positive psychology interventions based on the PERMA model in addition to standard care, and the control group (*n* = 60) that received standard care only. After a three-month follow-up period, glycemic control indicators (including Glycosylated Hemoglobin (HbA1c), fasting blood glucose, and 2-h postprandial blood glucose), diabetes-related psychological distress scores, and self-management behavior scale scores were compared between the two cohorts.

**Result:**

Compared to the control group, the intervention group demonstrated significantly greater improvements in HbA1c (7.16 ± 0.59), fasting blood glucose (5.53 ± 0.72), and 2-h postprandial blood glucose (8.26 ± 0.94) (all *p* < 0.05). Additionally, the intervention group showed significantly lower scores for diabetes-related psychological distress and significantly higher scores across all dimensions of self-management behaviors (all *p* < 0.05).

**Conclusion:**

The PERMA model-based positive psychology intervention effectively alleviates psychological distress, enhances self-management capabilities, and improves glycemic control in patients with T2DM. It represents a valuable adjunctive approach to standard diabetes care.

## Introduction

Diabetes has become one of the major global public health issues, with type 2 diabetes accounting for over 90% of all cases (Li et al., 2020). Effective diabetes management extends beyond pharmacotherapy, demanding long-term lifestyle modifications and strict self-management in areas such as diet, exercise, blood glucose monitoring, and foot care (Forouhi et al., 2018). The considerable burden of these complex regimens often precipitates adverse psychological symptoms—including worry, fear, anger, frustration, depression, and anxiety—with reported incidence rates ranging from 30 to 70% (Reddy et al., 2013). This specific form of psychological distress has been formally defined as the diabetes-associated psychological burden (Hermanns et al., 2006).

Evidence suggests a significant correlation between this psychological burden and self-care behaviors (Wardian and Sun, 2014). Such distress can directly impair treatment adherence, thereby compromising glycemic control, exacerbating psychological symptoms, and diminishing overall quality of life (Abbas et al., 2023). With the growing adoption of the biopsychosocial model in clinical practice, psychological interventions have gained increasing emphasis (Bolton, 2023). Appropriately designed positive psychology interventions can effectively alleviate psychological distress, enhance treatment adherence, and improve the quality of life in diabetic patients (Schmidt et al., 2018).

Cognitive behavioral therapy (CBT) is widely recognized as the cornerstone of psychological intervention for patients with type 2 diabetes. Extensive empirical research has shown that traditional CBT effectively alleviates these negative psychological states by helping patients identify and reframe maladaptive thought patterns, thereby breaking the cycle of distress and non-adherence (Abbas et al., 2023; Safren et al., 2014). By addressing these cognitive deficits, CBT lays a critical foundation for improving self-care and clinical outcomes. However, traditional cognitive behavioral therapy primarily focuses on reducing psychopathology and negative emotions, whereas the lifelong nature of type 2 diabetes requires not only the alleviation of distress but also the ongoing cultivation of psychological resilience. Consequently, emerging therapeutic frameworks build upon cognitive behavioral therapy by incorporating strengths-based approaches, often conceptualized as “positive cognitive behavioral therapy” (Padesky and Mooney, 2012). Our research is grounded in cognitive behavioral therapy (CBT) and utilizes a positive psychology framework, particularly the PERMA model, to address psychological distress and adherence in type 2 diabetes by supplementing CBT with behavioral activation and cognitive restructuring.

In 2011, Seligman proposed the PERMA model, which encompasses five key elements of positive psychology: Positive emotions (P), Engagement (E), Relationships (R), Meaning (M), and Accomplishment (A) (Seligman, 2011). These elements are essential for overall well-being, happiness, and human flourishing. Each component is distinctly defined: Positive Emotion encompasses feelings of happiness and satisfaction; Engagement refers to deep involvement in activities that promote flow and diminished self-consciousness; Relationships emphasize the importance of high-quality social connections; Meaning derives from having a sense of purpose in life; and Accomplishment relates to making progress toward and achieving meaningful goals (Farmer and Cotter, 2021; Seligman et al., 2006). This model has been demonstrated to have efficacy in enhancing positive emotions, promoting health-related behaviors, and improving life quality and prognosis (Fang et al., 2023).

Research shows that positive psychological resources (optimism, positive emotions, and well-being) can improve adherence through mediating factors such as self-efficacy and coping, and that PERMA-based interventions are feasible and well-received (Huffman et al., 2015). By shifting the therapeutic paradigm from traditional, deficit-based symptom relief to the proactive cultivation of positive psychological capital, interventions based on the PERMA model can effectively alleviate diabetes-related stress and may influence long-term adherence among people with diabetes (Kefei, 2025). The ongoing daily demands of managing type 2 diabetes (T2D) often lead to diabetes burnout, anxiety, and depression, all of which are major contributors to poor adherence to medication and dietary regimens (Hoogendoorn et al., 2024). However, by applying the PERMA framework, patients can break this negative cognitive cycle. Cultivating positive emotions can enhance the use of adaptive coping strategies, thereby alleviating the psychological burden of the illness (Kruger et al., 2023). In addition, by finding personal meaning in their self-care routines and acknowledging daily achievements, patients can develop a greater sense of self-efficacy; a recent systematic review confirmed that PERMA-based positive psychological interventions (PPIs) can significantly alleviate depression, enhance exercise efficacy, and improve overall self-management behaviors in adults with type 2 diabetes (Kefei, 2025). Ultimately, by prioritizing overall mental wellbeing rather than focusing solely on symptom management, the PERMA model may help enhance intrinsic motivation and psychological resilience, which in turn can support the maintenance of healthy behaviors and potentially improve clinical outcomes.

Nevertheless, applications of PERMA-based positive psychology interventions in T2DM management remain relatively unexplored. Therefore, this study aims to implement a PERMA model-based positive psychological intervention for type 2 diabetes patients and evaluate its effects. Given ethical and practical constraints of randomizing type 2 diabetes patients to psychological interventions during routine clinical care, this study employed a nonequivalent groups quasi-experimental design—an established methodology for evaluating interventions in real-world settings where randomization is infeasible (Arévalo Avalos et al., 2024).

## Methods

### Study design

This study was a single-center, quasi-experimental study conducted at Renmin Hospital of Wuhan University between January and December 2023. A total of 120 eligible patients with type 2 diabetes were enrolled. Participants were assigned to two non-equivalent groups based on clinical treatment pathways. The intervention group (*n* = 60) received an 8-session PERMA model-based positive psychology intervention plus standard diabetes care; the control group (*n* = 60) received standard care only (routine diabetes education and nursing support). Baseline data for all enrolled patients were systematically collected and recorded at the beginning of the study. Subsequently, both groups were followed for a period of 3 months. The main observation indicators included changes in glucometabolic parameters, diabetes-related distress scores, and self-management behavior scale scores, in order to evaluate the long-term effects of this intervention model on psychological distress and adherence behavior in patients. The study protocol received ethical approval from the Ethical Committee of the Renmin Hospital of Wuhan University (Approval No: WDRY2023-K045) and was conducted in accordance with the principles of the Declaration of Helsinki and the Medical Research Involving Human Subjects Act. Written informed consent was obtained from all patients. All PERMA interventions were conducted in a dedicated consultation room within the Endocrinology Department of Renmin Hospital of Wuhan University. The interventions and data collection took place after the COVID-19 pandemic was officially declared over, and standard hospital infection control protocols were maintained throughout the entire process.

### Participants

**Figure 1:**
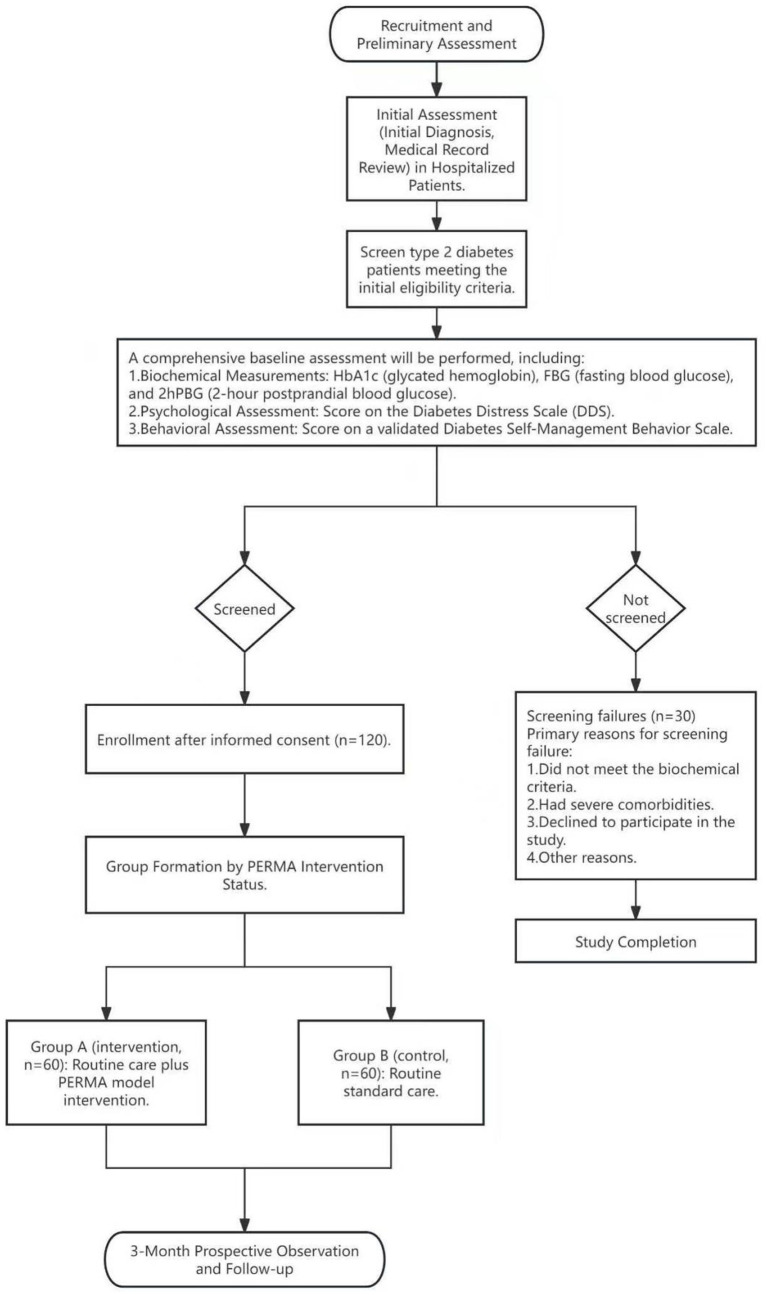
Research process flowchart.

Hundred and twenty diagnosed T2DM patients admitted to the Department of Endocrinology from January to December 2023 were selected. The selection criteria were as follows: ① Met the diagnostic criteria for T2DM specified in the “Chinese Guidelines for the Prevention and Treatment of Type 2 Diabetes (2020 Edition)”; ② Possessed independent living ability and were aged 18 or above; ③ Patients were required to complete the entire treatment course according to the study’s protocol and provide follow-up data within the specified period; ④ Patients and their families were informed and voluntarily participated in the study. Exclusion criteria: ① Patients unable to complete the intervention program provided by the hospital; ② Had psychiatric diseases, or impairments in consciousness, cognitive function, or language communication; ③ Had received other psychological therapies; ④ Suffered from other major internal diseases or had severe complications that might affect the study. Figure 1 illustrates our research process.

### Interventions

#### Control group (Group B)

The control group received routine care alongside systematic medical treatment. This standard nursing protocol encompassed diabetes-related health education delivered through multiple methods, including ward educational video screenings, distribution of health guidance materials, and individual face-to-face instruction to educate patients and their families about diabetes knowledge and health education. Content included: understanding the hazards of diabetes, medication guidance for diabetes, teaching patients self-monitoring and recording of blood glucose, recommendations for a scientific diet and regular aerobic exercise, providing psychological counseling, addressing psychological barriers, and encouraging patients to face their condition positively.

#### Intervention group (Group A)

In addition to standard treatment, the intervention group received a PERMA model-based positive psychology intervention. This intervention was delivered in eight sessions, scheduled twice weekly over 1 month, with each session lasting 30–60 min. The specific implementation procedure was as follows:

Formation of the Intervention Team: A dedicated positive psychology intervention team was established. All members were certified professional psychological consultants. A national-level psychological consultant led the training in intervention techniques and was responsible for overall quality control. The rest of the team was tasked with subject recruitment, condition monitoring, intervention implementation, and data collection and management.

Development of the Intervention Protocol: The team conducted an extensive review of pertinent literature and foundational works on positive psychology and the PERMA model. Through collaborative discussions, a preliminary intervention protocol tailored for T2DM patients was drafted. This draft was subsequently reviewed and refined by a panel of psychology experts, resulting in the final intervention protocol detailed in Table 1.

**Table 1:** PERMA model positive psychological intervention plan.

Session	Location	Theme	Method	Specific nursing measures	Technology type	Purpose
1	Ward	Positive cognition of self and disease	One-on-one interview	The intervener actively communicates with the patient to fully understand their perception of the disease and psychological acceptance; sincerely and patiently answers the patient’s questions, encouraging them to express their true feelings; uses non-verbal communication to promote emotional connection with the patient, building a solid trust bridge; invites optimistic fellow patients to share success stories, boosting the patient’s confidence.	Cognitive-affective behaviors	Establish trust with the patient and build a peer support system.
2	Ward	Actively establishing rational beliefs	One-on-one interview	Engage in conversation about the patient’s recent negative emotions; use a Q&A outline to guide the dialogue, correct misunderstandings about diabetes-related complications, and deepen diabetes prevention education. Q&A outline: What adjustments have you made in your daily attitudes and habits since being diagnosed with diabetes and experiencing related complications? What knowledge do you have about the oral hypoglycemic drugs or insulin you are taking? Have you followed a personalized diet plan according to medical advice for your health condition?	Cognitive-affective behaviors	Correct irrational beliefs and guide patients to establish rational beliefs.
3	Ward	Cultivating positive qualities	One-on-one interview Implementing “Three Good Things”	Conduct interviews focusing on people and things to be grateful for daily, share others’ gratitude events, discuss the benefits of gratitude, guide patients to think about things they are grateful for in life, provide patients with a pen and notebook, encourage them to record “Three Good Things” before bed each day, and bravely express thanks.	Cognitive-affective behaviors	Help discover the good things in life, cultivate a quality of gratitude, and encourage the expression of thanks.
4	Ward	Positive emotions	One-on-one interview	Introduce the important role of various positive emotions to the patient, guide the patient to recall scenes associated with each positive emotion, cultivate positive thinking, improve positive attitudes toward things, and encourage patients to use positive language to improve their own emotions, such as “I can,” “I am capable.”	Cognitive-affective behaviors	Train positive thinking, improve positive attitude, maintain positive and optimistic emotions.
5	Ward reading area	Engagement	One-on-one interview Participating in activities	Discuss topics related to engagement with the patient, guide the patient to understand the concept and sources of ‘flow’, based on the patient’s condition and personal preferences, carry out interesting activities such as: reading, playing chess, listening to music, yoga, aerobics, etc.	Motor behaviors	Distract the patient’s attention from their own illness.
6	Ward	Positive relationships	Interview role-playing	Explain the concept of active constructive responding to the patient, and guide the patient to participate in demonstrating the differences between active constructive, passive constructive, active destructive, and passive destructive response modes. Further explore the deeper meaning of interpersonal communication, coach patients in practical skills for interpersonal communication, encourage them to adopt active constructive responding, thereby changing their passive and negative coping attitudes. Additionally, organize patients and their families to participate in interactive role-playing themed around active responding to enhance the patient’s understanding of this concept.	Motor behaviors	Help patients actively constructively respond, and appreciate the benefits of positive communication methods.
7	Ward	Meaning	One-on-one interview	Discuss the value of life meaning with the patient, guide the patient to treat life with an optimistic mindset, help the patient recognize that the occurrence of the disease does not affect the definition of life meaning, thereby weakening the patient’s association between disease recovery and life meaning, truly learning to fly against the wind.	Cognitive-affective behaviors	Establish a positive concept of life meaning.
8	Ward	Achievement	One-on-one interview “Five Carriages”	Interview the patient about “achievement,” guide the patient to derive positive psychological effects from success, and encourage the patient to establish their own “Five Carriages” to achieve diabetes control goals, enhancing the patient’s sense of achievement by reaching target blood glucose levels.	Motor behaviors	Gain a sense of achievement.

### Procedure

Figure 1 summarizes the participant progression throughout this quasi-experimental study. Potential participants were screened for eligibility upon hospital admission. Those who met the inclusion criteria and provided written informed consent underwent a comprehensive baseline assessment (T0). During this assessment, all demographic, clinical, and outcome measure data were collected.

Subsequently, the participants were assigned to two groups based on the treatment pathways they subsequently followed in clinical practice. The intervention group (*n* = 60) consisted of patients who received the PERMA - based positive psychology intervention (a 1–month, 8–session program) in addition to standard care. The control group (*n* = 60) comprised patients who received standard care only, which included routine diabetes education and nursing support. All participants continued their prescribed pharmacological treatments as managed by their clinical team.

All enrolled patients were followed prospectively. A follow–up assessment (T1) was conducted 3 months after the baseline assessment. During this follow–up, all outcome measures were repeated by the research assessors.

### Assessment

#### Glucose metabolism indicators

Collected from both groups before and after the intervention, including HbA1c, FBG, and 2hPBG.

#### Diabetes distress scale (DDS)

The DDS assesses patients’ worries related to the disease and its self-management (Schmitt et al., 2015), used to measure the psychological distress level of diabetic patients. It has been proven by relevant scholars to be a good marker scale for assessing the quality of life of diabetic patients (Tanenbaum et al., 2016). Chinese scholar Zhang Yuyun revised and validated the scale (Zhang et al., 2022). The DDS contains 17 items covering four main aspects: emotional burden, physician-patient communication, life regimen, and social distress. Fisher et al. (2012) updated the scoring criteria, setting an average score of 2.0 as the cutoff, below 2.0 indicating no distress, 2.0 to 3.0 moderate distress, and above 3.0 severe distress. This study used Fisher’s scoring criteria. DDS performs well in many key indicators such as content validity, structural validity and reliability, which proves that it is a reliable and effective pain assessment tool for diabetes in China’s adult patients with type 2 diabetes (Zhang et al., 2022).

#### The summary of diabetes self-care activities (SDSCA)

SDSCA was developed by Toobert et al. (2000) and later introduced domestically by Sun Shengnan and Dong Yingyue (2011). The scale consists of 11 items forming 5 dimensions: foot care (2 items), blood glucose monitoring (2 items), exercise management (2 items), diet management (4 items), and smoking (1 item). The score for each item is primarily based on the number of days in the past week the patient was able to adhere to each dimension. Studies have shown that higher scores on this scale indicate stronger self-management ability and higher patient compliance (Toobert et al., 2000). The SDSCA has good validity and reliability, and the Cronbach’s alpha of the diet, exercise, blood sugar testing and foot care subscales ranged from 0.62 to 0.98 (Tang et al., 2021).

### Sample size calculation

This study employs a prior-based sample size estimation method. The primary outcome was designated as the change in the total score of the Diabetes Distress Scale (DDS) after the 3-month follow-up. The DDS was selected due to its established reliability and validity in assessing diabetes-specific psychological burden in Chinese populations with type 2 diabetes, with Cronbach’s *α* coefficients ranging from 0.76 to 0.81 for the total scale and its subscales as reported in prior validation studies (Zhang et al., 2022)。.

Based on a review of relevant literature on psychological interventions for T2DM, the minimal clinically important difference (MCID) for the DDS total score was set at 0.5 points (Fisher et al., 2012). The anticipated common standard deviation (SD) for the score change was estimated to be 1.0 point, derived from a preliminary analysis of our patient data and consistent with observed variability in similar studies.

Using G*Power software (version 3.1.9.7) for a two-tailed independent samples t-test, with an alpha (*α*) level of 0.05 and a desired statistical power (1 – *β*) of 80%, the calculation indicated a requirement of 64 participants per group to detect the specified effect size (Cohen’s *d* = 0.5). To account for a potential attrition rate of up to 15% during the follow-up period, the target sample size was inflated to 76 participants per group (total *N* = 152).

Considering practical recruitment constraints as a single-center study within 1 year, a final sample of 120 eligible participants (60 per cohort) was consecutively enrolled. While this number is slightly below the attrition-adjusted target, a post-hoc power analysis confirmed that the achieved sample of 60 per group provides 80% power to detect an effect size of *d* = 0.5 (the pre-specified MCID-based effect) at *α* = 0.05, which is adequate for the primary analysis. This sample size also affords sufficient power for secondary analyses on key metabolic outcomes (e.g., HbA1c).

### Statistical methods

All analyses were performed based on the intention-to-treat method. Data entry and statistical analysis were performed using SPSS 22.0 software (IBM, Armonk, NY, USA). Descriptive statistics were applied to sociodemographic and clinical characteristics. Quantitative data were expressed as mean ± standard deviation (x ± s) and analyzed using *t*-tests. Qualitative data were presented as frequency and percentage, and assessed using chi-square tests. A *p*–value of < 0.05 at the 95% CI was considered statistically significant.

## Results

This study included a total of 120 patients, with 60 in the experimental group (Group A) and 60 in the control group (Group B). As shown in Table 2, a comparative analysis was conducted on the demographic and clinical baseline data of the two groups of patients before intervention to ensure comparability between the two groups. There were no significant differences in baseline characteristics between the two groups (*p* > 0.05).

**Table 2:** Comparison of general data between the two groups of patients (x̄ ± s).

Indicator	Intervention Group (Group A, *n* = 60)	Control Group (Group B, *n* = 60)	*t*	*P*
Gender (Male/Female)	31/29	32/28	0.330	0.86
Age (years)	46.17 ± 11.69	46.83 ± 12.45	0.302	0.41
Education level (High school and below/College and above)	32/28	34/26	0.724	0.39
Marital status (With/Without spouse)	55/5	52/8	0.849	0.36
Occupation (Employed/Unemployed)	48/12	44/16	0.976	0.32
Diabetes duration (years)	5.15 ± 2.87	5.97 ± 3.22	1.466	0.37
Payment method (Medical insurance/Self-pay)	55/5	55/5	3.841	1

### Changes in glucose metabolism indicators before and 3 months after intervention in both groups

Before the intervention, the differences in HbA1c, FBG, and 2hPBG data between the two groups were not statistically significant. After the intervention, glucose metabolism indicators decreased in both groups. The improvement in the three glucose metabolism indicators in the experimental group after the PERMA-based positive psychology intervention was better than that in the routine group, and the improvements in these indicators showed statistically significant differences, as detailed in Table 3.

**Table 3:** Comparison of glucose metabolism indicators before and 3 months after intervention between the two groups (x ± s).

Indicator	*n*	Fasting blood glucose (mmol/L)			2-h postprandial blood glucose (mmol/L)			Glycated hemoglobin (%)		
		Pre-intervention	Post-intervention	*t*(p)	Pre-intervention	Post-intervention	*t*(p)	Pre-intervention	Post-intervention	*t*(p)
Intervention group	60	13.25 ± 2.47	5.53 ± 0.72	24.63 (<0.05)	17.02 ± 1.50	8.26 ± 0.94	34.37(<0.05)	9.98 ± 0.92	7.16 ± 0.59	18.67(<0.05)
Control group	60	13.95 ± 2.25	7.24 ± 0.89	47.87 (<0.05)	16.77 ± 1.71	8.44 ± 0.86	36.28(<0.05)	10.24 ± 1.11	7.90 ± 0.65	20.52(<0.05)
*t*	–	1.46	11.24	–	0.87	1.10	–	1.42	6.73	–
*P*	–	>0.05	<0.05	–	>0.05	>0.05	–	>0.05	<0.05	–

### Comparison of diabetes-related distress scores before and 3 months after intervention in both groups

Before the intervention, there were no significant statistical differences in the scores across various dimensions of diabetes-related distress between the two groups (*p* > 0.05). However, after the intervention, the scores of the experimental group on all dimensions of the Diabetes Distress Scale were significantly lower than those of the routine group, and these differences reached statistical significance, as detailed in Table 4.

**Table 4:** Comparison of diabetes-related distress scores before and 3 months after intervention between the two groups (x ± s).

Group	*n*	Total psychological distress score	Emotional burden-related distress	Life regimen-related distress	Interpersonal distress	Physician-related distress
Pre-intervention						
Intervention Group	60	3.34 ± 0.20	2.89 ± 0.33	3.74 ± 0.43	3.66 ± 0.48	3.14 ± 0.41
Control Group	60	3.37 ± 0.22	2.92 ± 0.27	3.81 ± 0.49	3.64 ± 0.40	3.19 ± 0.38
*t*	-	−0.78	−0.55	−0.83	0.25	−0.69
*P*	-	>0.05	>0.05	>0.05	>0.05	>0.05
Post-intervention						
Intervention Group	60	1.98 ± 0.14	2.04 ± 0.18	2.07 ± 0.30	1.81 ± 0.37	1.94 ± 0.27
Control group	60	2.46 ± 0.16	2.29 ± 0.24	2.62 ± 0.35	2.52 ± 0.31	2.43 ± 0.36
*t*	–	−17.52	−6.46	−9.24	−11.39	−8.43
*P*	–	<0.05	<0.05	<0.05	<0.05	<0.05

### Comparison of diabetes self-management behavior scale scores before and 3 months after intervention in both groups

Before the intervention, there was no statistically significant difference in the self-management level between the two groups (p > 0.05). After the intervention, the scores in all aspects of self-management in the experimental group significantly increased compared to before the intervention. Furthermore, the self-management ability scores of the experimental group were significantly higher than those of the routine group (*p* < 0.05). All between-group comparisons yielded statistically significant results, as detailed in Table 5.

**Table 5:** Comparison of diabetes self-management behavior scale scores before and 3 months after intervention between the two groups (x ± s).

Group	*n*	Total self-management score	Diet management	Exercise management	Blood glucose monitoring	Foot care
Pre-intervention						
Intervention Group	60	8.73 ± 1.56	3.30 ± 0.48	3.52 ± 1.13	1.33 ± 0.85	0.59 ± 0.61
Control Group	60	8.82 ± 1.79	3.26 ± 0.50	3.68 ± 1.45	1.23 ± 1.08	0.65 ± 0.58
*t*	—	0.29	0.44	−0.67	0.59	−0.52
*P*	—	>0.05	>0.05	>0.05	>0.05	>0.05
Post-intervention						
Intervention group	60	21.93 ± 1.66	5.51 ± 0.41	5.40 ± 0.76	4.63 ± 0.86	6.38 ± 0.80
Control group	60	16.30 ± 1.62	4.69 ± 0.41	4.61 ± 1.14	3.42 ± 0.72	3.58 ± 0.77
*t*	—	18.44	10.73	4.53	8.43	20.29
*P*	—	<0.01	<0.01	<0.05	<0.05	<0.05

## Discussion

### Positive psychology intervention based on the PERMA model helps improve glucose metabolism indicators in T2DM patients

When patients with type 2 diabetes (T2DM) experience prolonged hyperglycemia, this condition can lead to endocrine and metabolic disorders, triggering a variety of serious acute and chronic complications that can even be life-threatening in severe cases. The most direct indicator for describing the control of T2DM is the level of blood glucose control (Grady et al., 2015), and HbA1c can reflect blood glucose control over the past 2–3 months. Achieving target levels of blood glucose and HbA1c is the treatment goal for diabetes. Research by Ogbera found a positive correlation between T2DM-related psychological distress and glucose metabolism (Ogbera and Adeyemi-Doro, 2011).

The results of this study indicate that positive psychology interventions based on the PERMA model have a significant impact on objective clinical indicators. Three months after the intervention, the fasting blood glucose (FBG), 2-h postprandial blood glucose (2hPBG), and glycated hemoglobin (HbA1c) levels in the experimental group were significantly lower than those in the conventional group, and the differences between the groups were statistically significant. The mechanism by which psychological changes translate into physiological health can be understood through two primary and intertwined pathways: behavioral maintenance and neuroendocrine regulation. At the behavioral level, actively cultivating the PERMA pillars—particularly a sense of meaning and a sense of accomplishment—can foster the high self-efficacy and intrinsic motivation necessary to sustain rigorous lifestyle changes (Ibrahim et al., 2023). When patients transition from a mode of compulsory compliance to meaningful, goal-oriented self-care, they demonstrate greater consistency in their nutritional management and physical activity. These sustained behavioral changes will lead to improvements in metabolic parameters, including fasting blood glucose (FBG), 2-h postprandial blood glucose (2hPBG), and hemoglobin A1c (HbA1c) levels.

At the same time, there may be a biological link between psychological distress and glycemic control via neuroendocrine pathways. Studies have shown that psychological distress in chronic diabetes is characterized by sustained activation of the hypothalamic–pituitary–adrenal (HPA) axis and the sympathetic nervous system, leading to elevated circulating cortisol levels and systemic inflammation (Witek et al., 2019). These stress-induced physiological states significantly exacerbate insulin resistance and stimulate hepatic gluconeogenesis. By fostering positive emotions and strengthening interpersonal relationships, PERMA-based interventions effectively buffer this chronic stress response (Yaribeygi et al., 2022), thereby reducing cortisol and inflammatory cytokines, biochemically improving cellular insulin sensitivity, and promoting reductions in fasting blood glucose (FBG) and glycated hemoglobin (HbA1c) levels (Ortiz et al., 2019). Therefore, by addressing patients’ psychological and emotional well-being, the PERMA model can serve as a powerful catalyst for optimizing blood glucose levels.

### Positive psychology intervention based on the PERMA model helps reduce psychological distress in T2DM patients

This study revealed no significant difference in psychological distress scores between the two groups at baseline. Following the implementation of the PERMA-based positive psychology intervention, however, the psychological distress score of the intervention group was significantly lower than that of the control group, suggesting the efficacy of this approach in alleviating psychological distress among T2DM patients. This therapeutic effect may be attributed to the PERMA model’s capacity to enhance patients’ psychological resources across multiple dimensions. Existing evidence indicates that diabetic patients often experience psychological distress due to disease burden, management pressure, and lack of support (Stanković et al., 2013). Positive psychology interventions can facilitate patients’ accurate understanding of their condition, strengthen treatment confidence, ameliorate negative emotions, and enhance their role adaptation and self-management abilities (Ha et al., 2014), thereby comprehensively reducing psychological distress from cognitive, emotional, and behavioral levels. The incorporation of the PERMA framework into clinical practice can be realized through structured interventions: guiding patients to focus on positive emotions and build confidence in disease management; encouraging patients to engage in feasible self-care activities to enhance a sense of control; promoting supportive communication between patients and family, peers, and healthcare providers; helping them find meaning in disease management and establish feasible blood glucose control goals; promptly affirming their progress in treatment adherence and self-management to enhance a sense of achievement. This systematic psychological support approach can thereby enhance patients’ psychological adjustment and overall rehabilitation quality beyond conventional treatment alone.

### Positive psychology intervention based on the PERMA model helps improve compliance in T2DM patients

The PERMA model, a framework within positive psychology, formulates targeted psychological intervention strategies for clients through five core dimensions—Positive Emotion, Engagement, Relationships, Meaning, and Accomplishment. Through multiple in-depth interviews with patients and activities such as “Three Good Things” and “Role-Playing, “it promotes patients’ self-awareness of the disease, corrects erroneous beliefs about the disease, guides the establishment of rational beliefs, enhances positive emotions and a grateful disposition, maintains stable emotions, reduces diabetes-related psychological distress, improves quality of life, enhances compliance, and achieves blood glucose control goals. Strengthening self-management skills plays a significant positive role in the rehabilitation process of chronic diseases (Mackey et al., 2016).

In this study, a dedicated PERMA intervention team composed of senior psychological consultation was established. The team built a strong trust-based rapport with patients and guided them in deeply exploring and practicing aspects such as positive self-perception, emotional well-being, healthy social relationships, engagement, gratitude, goal pursuit, sense of accomplishment, and meaning in life. This process aimed to rectify maladaptive thinking patterns, cultivate positive emotions, and enhance patients’ recognition of the importance of a constructive mindset in managing disease progression, thereby effectively improving patients’ self-management ability and treatment compliance (Bernard et al., 2018).

Before the intervention, no statistically significant difference was observed in self-management scores between the two groups (*p* > 0.05). After the intervention, however, the intervention group showed significantly higher scores across all self-management dimensions compared to both their own baseline levels and those of the control group (*p* < 0.05). These results indicate that the positive psychology intervention based on the PERMA model has a beneficial effect on enhancing self-management behaviors and treatment adherence in patients with T2DM.

### Limitations

This study has several limitations that should be considered. First, the findings may have limited generalizability due to the relatively small sample size and the single-center design. Future multi-center studies with larger samples are needed to validate our results. Second, the 3-month follow-up period is relatively short; thus, the long-term sustainability of the observed benefits remains unknown, and longer-term assessments are warranted. Third, while outcome assessors were blinded, it was not feasible to blind participants or intervention facilitators due to the nature of the psychological intervention, which may have introduced potential performance or response biases. Fourth, we did not conduct a formal mediation analysis to empirically test the mechanisms through which the PERMA model exerts its effects. Fifth, this study did not use established criteria such as the Reliable Change Index and cutoff values proposed by Jacobson and Truax to assess clinical significance; these metrics could have clearly identified the proportion of patients who achieved reliable improvement and functional recovery. Future studies should incorporate these metrics alongside normative data on diabetes-specific distress and adherence measures to more accurately assess clinical significance. Finally, this quasi-experimental design carries selection bias as the primary threat to internal validity, since non-random assignment based on clinical pathways may result in systematic group differences. Although baseline characteristics were comparable and adjusted statistically, residual confounding remains possible. Future research should prioritize randomized designs while preserving real-world relevance through pragmatic trials and advanced causal inference methods.

## Conclusion

In summary, achieving glycemic targets in patients with T2DM extends beyond pharmacological treatment; it also necessitates a positive mindset and robust self-management capabilities. Compared to CBT, which aims to reduce symptoms through cognitive restructuring, the PERMA model actively fosters well-being in areas such as positive emotions, engagement, relationships, meaning, and accomplishment. By alleviating psychological distress in people with diabetes and strengthening their self-management skills and treatment adherence, it addresses the limitations of CBT in maintaining long-term motivation and resilience in chronic disease management. Therefore, this intervention represents a valuable and recommendable adjunct to standard diabetes care.

## Data Availability

The raw data supporting the conclusions of this article will be made available by the authors, without undue reservation.
